# Modeling total predation to avoid perverse outcomes from cat control in a data‐poor island ecosystem

**DOI:** 10.1111/cobi.13916

**Published:** 2022-08-24

**Authors:** Michaela Plein, Katherine R. O'Brien, Matthew H. Holden, Matthew P. Adams, Christopher M. Baker, Nigel G. Bean, Scott A. Sisson, Michael Bode, Kerrie L. Mengersen, Eve McDonald‐Madden

**Affiliations:** ^1^ School of Earth and Environmental Science University of Queensland St Lucia Queensland Australia; ^2^ Centre for Biodiversity and Conservation Science University of Queensland St Lucia Queensland Australia; ^3^ Administration de la nature et des forêts Diekirch Luxembourg; ^4^ School of Chemical Engineering University of Queensland St Lucia Queensland Australia; ^5^ School of Biological Sciences University of Queensland Brisbane Queensland Australia; ^6^ School of Mathematics and Physics University of Queensland Brisbane Queensland Australia; ^7^ School of Mathematical Sciences Queensland University of Technology Brisbane Queensland Australia; ^8^ ARC Centre of Excellence for Mathematical and Statistical Frontiers Queensland University of, Technology Brisbane Queensland Australia; ^9^ School of Mathematics and Statistics The University of Melbourne Parkville Victoria Australia; ^10^ Melbourne Centre for Data Science The University of Melbourne Parkville Victoria Australia; ^11^ Centre of Excellence for Biosecurity Risk Analysis The University of Melbourne Melbourne Victoria Australia; ^12^ School of Mathematical Sciences University of Adelaide Adelaide South Australia Australia; ^13^ Australian Research Council Centre of Excellence for Mathematical and Statistical Frontiers University of Adelaide Adelaide South Australia Australia; ^14^ School of Mathematics and Statistics University of New South Wales Sydney New South Wales Australia; ^15^ UNSW Data Science Hub University of New South Wales, Sydney New South Wales Australia

**Keywords:** ecosystem modeling, information scarcity, invasive species management, multiple threats, perverse consequences, amenazas múltiples, consecuencias accidentales, escasez de información, especie invasora, gestión, modelación de ecosistemas, 多重威胁, 生态系统建模, 信息缺乏, 入侵物种, 管理, 意外后果

## Abstract

Data‐hungry, complex ecosystem models are often used to predict the consequences of threatened species management, including perverse outcomes. Unfortunately, this approach is impractical in the many systems that have insufficient data to parameterize ecosystem interactions or reliably calibrate or validate such models. We devised a different approach composed of a minimum realistic model that guides decisions in data‐ and resource‐scarce systems. We applied our approach to a case study in an invaded ecosystem from Christmas Island, Australia, where there are concerns that cat (*Felis catus*) eradication to protect native species, including the red‐tailed tropicbird (*Phaethon rubricauda*), could release mesopredation by invasive rats (*Rattus rattus*). We used biophysical constraints (metabolic demand) and observable parameters (e.g., prey preferences) to identify the combined cat and rat abundances that could threaten the tropicbird population. The population of tropicbirds was not sustained when predated by 1607 rats (95% credible interval [CI]: 103–5910) in the absence of cats and 21 cats (95% CI: 2–82) in the absence of rats. For every cat removed from the island, the bird's net population growth rate improved, provided rats did not increase by more than 77 individuals (95% CI: 30–174). Thus, in this context, 1 cat is equivalent to 30–174 rats. Our methods are especially useful for on‐the‐ground predator control in the absence of knowledge of predator–predator interactions to determine whether current abundance of predators threatened the prey population of interest; managing only 1 predator species was sufficient to protect the prey species given potential release of another predator; and control of multiple predator species was needed to meet the conservation goal. With our approach limited information can be used for maximum value in data‐poor systems because it shifts the focus from predicting future trajectories to identifying conditions that impede conservation.

## Resumen

Los modelos ambientales complejos y con un gran volumen de datos se usan con frecuencia para pronosticar las consecuencias de la gestión de las especies amenazadas, incluyendo los resultados perversos. Desafortunadamente, esta estrategia no es práctica en los tantos sistemas que no tienen suficientes datos para formular un parámetro de las interacciones en el ecosistema o calibrar o validar con confianza dichos modelos. Diseñamos una estrategia diferente compuesta de un modelo realista mínimo que orienta las decisiones dentro de los sistemas con escasez de datos y recursos. Aplicamos nuestra estrategia a un estudio de caso en un ecosistema invadido de la Isla Navidad, Australia, en donde existe preocupación por la eliminación del gato doméstico (*Felis catus*) para proteger a las especies nativas, incluida el ave tropical de cola roja (*Phaethon rubricauda*), como posible impulsor de la mesodepredación por ratas invasoras (*Rattus rattus*). Usamos restricciones biofísicas (demanda metabólica) y parámetros observables (p. ej.: preferencia por presas) para identificar la abundancia combinada de gatos y ratas que podrían amenazar a la población de *Phaethon rubricauda*. La población de esta especie no fue constante cuando fue presa de 1607 ratas (95% intervalo de credibilidad [IC] 103–5910) en ausencia de gatos y cuando fue presa de 21 gatos (95% IC 2–82) en ausencia de ratas. Por cada gato que se eliminó de la isla, la tasa neta de crecimiento poblacional de la especie aumentó, bajo la condición de que las ratas no incrementaran en más de 77 individuos (95% IC 30‐174]). Por lo tanto, dentro de este contexto, 1 gato es equivalente a 30–174 ratas. Nuestros métodos son realmente útiles para el control in situ de depredadores en ausencia de información sobre las interacciones depredador‐depredador para determinar si la abundancia actual de depredadores amenaza a la población presa de interés. La gestión de 1 especie depredadora fue suficiente para proteger a la especie presa bajo la potencial liberación de otro depredador y se necesitó del control de varias especies depredadores para lograr el objetivo de conservación. Con nuestra estrategia, la información limitada puede usarse para el valor máximo en los sistemas deficientes de información ya que cambia el enfoque de la predicción de futuras trayectorias a la identificación de las condiciones que impiden la conservación.

## 在数据匮乏的岛屿生态系统中构建总捕食量模型以避免对猫的数量管理导致不当结果

**【摘要】** 需要大量数据的复杂生态系统模型经常被用于预测管理受威胁物种的结果, 包括反常结果。然而, 这种方法在许多系统中是不切实际的, 因为这些系统没有足够的数据来对确定生态系统互作的参数, 或是可靠地校准或验证这些模型。我们设计了一种由最小现实模型组成的新方法, 可以在数据和资源缺乏的系统中指导决策。我们将该方法应用于澳大利亚圣诞岛遭受生物入侵的生态系统的案例研究中, 当地为保护原生物种(包括红尾热带鸟[*Phaethon rubricauda*])而消灭猫(*Felis catus*)的举措可能会放松对入侵的中等捕食者老鼠(*Rattus rattus*)的压力, 引发了人们的担忧。我们使用生物物理限制(代谢需求)和可观测参数(如猎物偏好)确定了可能威胁到热带鸟种群的猫和鼠的综合丰度。在没有猫的情况下, 热带鸟会被1607只老鼠(95%置信区间[CI] 103‐5910)捕食, 而在没有老鼠的情况下, 会被21只猫(95% CI 2‐82)捕食, 种群都无法维持。每从岛上移除一只猫, 只要老鼠不增加超过77只(95% CI 30‐174), 鸟类的净增长速度就会提高。因此, 在这种情况下, 1只猫相当于30‐174只老鼠。我们的方法特别适用于以下情况:在不了解捕食者互作的情况下进行实地捕食者控制, 以确定当前的捕食者丰度是否会威胁到关注的被捕食者种群;考虑到对另一种捕食者潜在的捕食压力放松, 只管理一种捕食者就足以保护被捕食者物种;需要控制多种捕食者以达到保护目标。我们的方法将重点从预测未来轨迹转移到识别阻碍保护的条件, 因此可以在数据缺乏的系统中将有限的信息可以发挥最大价值。**【翻译:胡怡思;审校:聂永刚】**

## INTRODUCTION

Managing threatened species in the face of ecosystem complexity and uncertainty can result in unintended consequences that undermine conservation goals (Pearson et al., 2022; Shannon et al., 2009; Wittmer et al., 2013). A range of methods have been developed to predict these perverse management outcomes, mostly via modeling population dynamics and species interactions (Baker et al., 2017; Bode et al., 2015; Dambacher et al., 2003). Although such ecosystem models provide insights for comparing broad conservation policies (Adams et al., 2020; Bode et al., 2015; Perryman et al., 2021; Rendall et al., 2021; Reum et al., 2021) (e.g., whether to remove invasive predators or invasive competitors of a threatened species [Rendall et al., 2021]), they typically do not inform day‐to‐day operations for managers, especially in data‐poor systems. We devised an alternative approach that applies minimum realistic, biophysically constrained models to bridge the gap between policy and operational conservation decisions for ecosystem managers faced with the all‐too‐common situation of limited ecosystem information.

Our approach is best illustrated through the example of predator control. Invasive predators present one of the most important issues in conservation, contributing to 58% of all known bird, mammal, and reptile extinctions (Doherty et al., 2016). To prevent future extinctions, managers regularly implement programs to control populations of invasive predators (Smith et al., 2010). Unfortunately, predator control can lead to perverse outcomes, such as releasing mesopredators that then increasingly prey on the threatened species, undermining the efficacy of predator control (Richie & Johnson, 2009). Mesopredator release is notoriously difficult to predict, and although pervasive (Prugh et al., 2009), it is not ubiquitous (Jachowski et al., 2020). Classical ecosystem models have in some cases identified when predator control might succeed given the possibility of mesopredator release (Baker et al., 2020; Bode et al., 2015). However, to capture species interactions, ecosystem models require many parameters that are challenging to accurately quantify (Geary et al., 2020), some of which (e.g., per capita interaction strengths) are difficult to interpret and measure (Baker et al., 2018). Furthermore, ecosystem models typically predict population trajectories under various scenarios, usually focused on a subset of management options (e.g., Adams et al., 2020; Peterson et al., 2021), rather than identifying the conditions under which conservation targets are at threat. This latter aim is unlikely to be achievable in the common situation in which missing data prevent adequate parameterization of these ecosystem models. Thus, there is a need to inform conservation decisions such that the potential for perverse outcomes is avoided without the need for exhaustive, complex ecosystem data that are often expensive and time‐consuming to collect and difficult to interpret. So, what should managers use when they need to make a quick decision in an ecosystem with the potential for perverse outcomes but do not have enough data to predict outcomes with ecosystem models?

We propose that minimum realistic models can address this need because they provide a useful path between the twin perils of ignoring ecosystem complexity and requiring extensive and expensive site‐specific data for parameterization. Minimum realistic models model the fewest ecosystem components and processes directly related to the model objective (Geary et al., 2020) and are particularly valuable if their parameters and outputs can be estimated quantitatively or qualitatively by managers. For example, instead of predicting ecosystem trajectory under different scenarios, a minimum realistic model can quantify the increase in mesopredators that would eliminate a conservation gain associated with invasive predator control.

**FIGURE 1:**
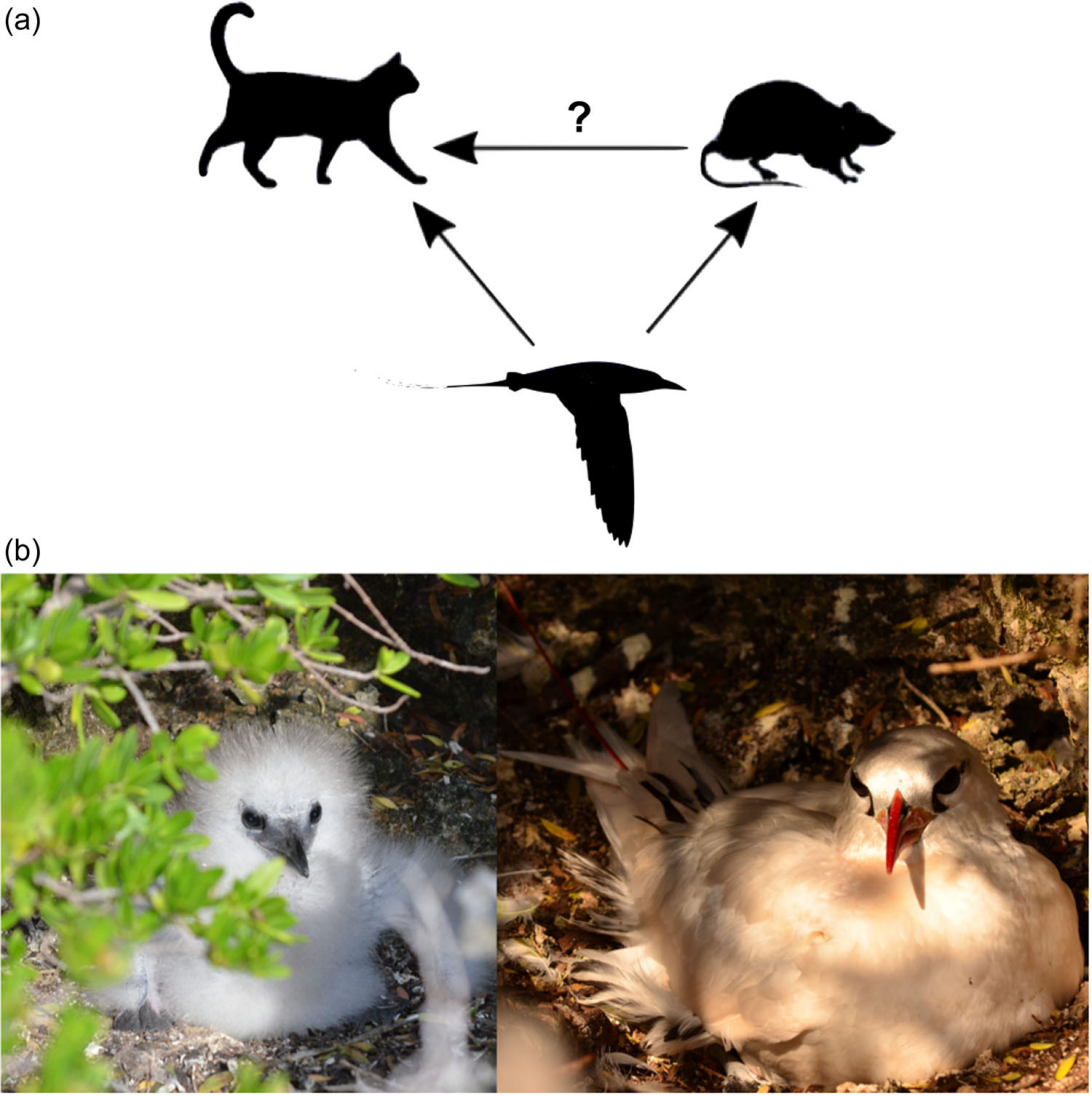
(a) The trophic network comprising feral cats, invasive black rates, and native red‐tailed tropic birds on Christmas Island (arrows, flow of energy; question mark, mesopredator release unknown). (b) Red‐tailed tropicbirds: hatchling (left) (photo by M. Plein) and breeding adult (right) (photo by M. Plein)

We applied our approach to a case study of an invaded ecosystem on Christmas Island, where habitat for threatened species is challenging to monitor due to the island's terrain and limited resources. On the island, feral cats (*Felis catus*) and invasive black rats (*Rattus rattus*) prey on the threatened red‐tailed tropicbird (*Phaethon rubricauda*) among other species (Beeton et al., 2010) (Figure 1). Cat removal is already underway, but there is concern that a decrease in the cat population could release predation pressure on rats, potentially leading to increased rat predation on red‐tailed tropicbirds (*Phaethon rubricauda*) (Baker et al., 2020; Han et al., 2020). Simultaneous rat control has therefore been recommended (Han et al., 2020). However, given the uncertainty associated with mesopredator release and the costs and difficulties of rat eradication on tropical islands (Holmes et al., 2015), complete rat control is unrealistic in this case. To provide advice for identifying and responding to mesopredator release, we used a minimum realistic model to calculate critical abundances of cats and rats that would yield population decline for red‐tailed tropicbirds. That is, rather than predicting the trajectories of individual species, we identified the degree of mesopredator release that would undermine tropicbird conservation. Because rat and cat population sizes are easy to interpret and can be assessed either qualitatively or quantitatively, thresholds of concern for these quantities can directly inform conservation. Identification of threshold population sizes for the more easily measured predators is especially useful because the prey species are often difficult to accurately monitor directly.

To generate critical predation thresholds, our minimum realistic model calculated the effects of rat and cat predation on the long‐term stability of the red‐tailed tropicbird population through an indicator metric, the reproduction ratio. To overcome the lack of system‐specific data, we combined biophysical constraints (e.g., metabolic demand) with information about the tropicbird's life cycle and predator impacts at different life stages. Through our approach, we estimated the levels of predation that are acceptable or unacceptable for the threatened species. Our approach allowed expression of the impact of 1 predator in terms of the other—the number of rats that have the same negative impact on the tropicbird population as 1 cat (a metric we call *cat equivalence*) to offer insight into the predator's relative impact on the conservation target species. This can support prioritization decisions about which predators to mitigate first or most intensively. Finally, we specifically included variability and uncertainty in model parameters and propagated these uncertainties through to predictions so that decision makers could explore their own risk tolerance and make more transparent decisions.

## METHODS

### Case study

Christmas Island is an Australian territory of 135 km^2^ in the Indian Ocean. Feral cats and black rats arrived at the island more than 100 years ago. Since then, both species have become invasive, and they threaten a number of native species, including red‐tailed tropicbirds (Beeton et al., 2010; Ishii, 2006). Due to the high risk posed by these invaders, a cat eradication program commenced on the island in 2010 with plans for a rat eradication program to follow (Algar & Hamilton, 2014). Because cats also eat rats, the 2 threats can interact antagonistically (i.e., the predation by rats may be lower in the presence of cats [Hughes et al., 2019; Rayner et al., 2007]). Controlling cats therefore has the potential to cause perverse consequences for native species if the reduction of cats leads to a mesopredator release of rats (Baker et al., 2018; Beeton et al., 2010; Han et al., 2020).

Red‐tailed tropicbirds are medium‐sized seabirds occurring on islands and coastal regions of the tropics in the Indian and Pacific Oceans (Fleet, 1974). Although red‐tailed tropicbirds spend most of their lives at sea, they nest on the ground (Figure 1), where they are vulnerable to terrestrial predation. After the juveniles fledge, all birds leave the island and spend their time offshore foraging until they return the next year to breed. Juvenile birds stay out to sea until they reach reproductive maturity (Fleet, 1974). Each breeding pair typically produces 1 egg per season until the end of their reproductive life span, around 13−16 years (Schreiber, & Schreiber, 1993). Although the current population size is unknown, historic estimates range from 1440 to 2000 breeding pairs (James et al., 2014; Stokes, 1988). Monitored breeding success on Christmas Island has been very low over the past 30 years, likely due to predation by non‐native species (Hennicke & Flachsbarth, 2009; Ishii, 2006; Sommerfeld et al., 2015). Unfortunately, limited resources (e.g., personnel, funding) and high environmental complexity (e.g., dense rainforest vegetation, sharp cliffs) on Christmas Island pose major constraints on assessment and management of threatened species and their threats. Managers may therefore not detect a decline in the red‐tailed tropicbird population until too many individuals have been lost for recovery of the population.

### Model overview

To assess the level of predation pressure by cats and rats on the population of red‐tailed tropicbirds, we developed a minimum realistic model that described the long‐term stability of the red‐tailed tropicbird population through an indicator metric. This indicator accounted for the bird's birth rate, natural mortality, and the mortality due to predation by cats and rats. Crucially, we considered the predation ratio by cats and rats at different stages of the seabird's life cycle: although rats consume eggs and hatchlings (juvenile birds in nests), cats prey on hatchlings and adult birds. We assumed the daily predation from cats and rats to be constant throughout the bird's breeding period on the island (approximately 2.5–3.0 months). The indicator accounted for short‐term changes in the adult bird population, due to direct cat predation of adults in the breeding season, and for long‐term impacts through egg and hatchling predation by rats. All calculations were performed in Matlab (Matlab, 2021).

### Model structure

We defined the persistence indicator as the reproduction ratio, *η*, which was the number of juvenile birds that hatched in the current breeding season and survived to reproductive maturity at 3 years of age (*N*_J,3_), compared with the loss in adult bird population over the current breeding season (i.e., difference between the adult population at the start [*N*_A,0_] and end [*N*_A,1_] of the breeding season):

(1) $$\eta =\frac{N_{J,3}}{N_{A,0}-N_{A,1}}.$$

Hence, if *η* < 1, the mortality rate of breeding birds is larger than the number of birds hatched that season expected to survive to breeding age, causing the population of red‐tailed tropicbirds to decline over time. Conversely, *η* > 1 indicates that the tropicbird population increases over time.

Because the 2 predator species affect the life stages of red‐tailed tropicbirds (e.g., adult birds, eggs, and chicks) differently and we lacked detailed information to parameterize a full life‐cycle population model, finding a suitable persistence indicator for the threatened species was a key challenge. An indicator that accounted for only the change in adult bird population would have failed to capture the effects of breeding failures through predation on eggs and hatchlings, until such a change finally manifested in the size of the adult breeding population. Conversely, focusing on breeding success would have omitted the predation of the adult population.

We calculated the change in adult population over the first breeding season, *N*_A,0_ − *N*_A,1_, based on the adult bird population at the start of the breeding season *N*_A,0_, an annual natural adult mortality *μ*_A_, the size of the cat populations *N*_cats_, the average duration of the breeding season *T*_B_, during which time the birds were exposed to predation, and the number of adult birds consumed per cat per day *p*_A,C_ as:

(2) $$N_{A,0}-N_{A,1}=N_{A,0}\mu_{A}+N_{\mathrm{cats}}p_{A,C}T_{B}.$$

The number of juveniles hatching in year 1, *N*_J,1_, that survive to reproduce after year 3, *N*_J,3_, depends on the initial population of breeding birds, *N*_A,0_; number of eggs laid per adult bird, *β*; proportion of eggs that hatch, *ν*; number of eggs eaten per rat per day, *p*_E,R_; number of hatchlings (juvenile birds after hatching and before leaving the nest) eaten per cat and rat per day *p*_H,C_ and *p*_H,R_, respectively; egg incubation time *T*_I_; and time hatchlings spend in the nest, *T*_H_, where breeding time *T*_B_ = *T*_I_ + *T*_H_:

(3) $$\begin{array}{ccc} N_{J,3} & = & \left[\nu \left(\beta N_{A,0}-N_{\mathrm{rats}}P_{E,R}T_{I}\right)\left(1-\mu_{H}\right)-N_{\mathrm{cats}}p_{H,C}T_{H}\right.\\ & - & {\left.N_{\mathrm{rats}}p_{H,R}T_{H}\left(1-\mu_{J}\right)\right]}^{T\mathrm{mat}}. \end{array}$$

Equation (3) arises from a mass balance of the juvenile population until they become reproductively mature, *N*_J,3_, adjusted for the initial hatchling mortality (1 − *μ*_H_), and fledgling mortality (juveniles after leaving the nest and before becoming reproductively mature) (1 − *μ*_J_)*^T^* ^mat^. Here, *μ*_J_ describes the overall annual juvenile mortality and *T*_mat_ refers to the time that juveniles spend off the island until they become reproductively mature. To avoid negative abundances of red‐tailed tropicbirds, the following constraints were included in Equations (1) and (2) in the MATLAB code: (1 – *μ*) *N*_A,0_ – *N*_cats_ *p*_A,C_ *T*_B_ ≥ 0 (i.e., cat predation of adult birds cannot exceed adult bird population); *β* *N*_A,0_ – *N*_rats_ *p*_E,R_ *T*_I_ ≥ 0 (i.e., rat predation of eggs cannot exceed the number of eggs laid); and *ν*(*β* *N*_A,0_ – *N*_rats_ *p*_E,R_ *T*_I_) – *p*_H,R_ *N*_rats_ *T*_H_ – *p*_H,C_ *N*_cats_ *T*_H_ ≥ 0 (i.e., combined cat and rat predation of hatchlings cannot exceed the number of eggs hatched).

### Predation rates

Predation rates of cats and rats on different life stages of red‐tailed tropic birds (in units of prey per day and predator individual) were calculated from the energetic demand of predators (metdemand_C_ and metdemand_R_ in Joules per day and predator individual); energy contents of prey items (energy_E_, energy_H_, and energy_A_ in Joules per gram and prey individual); masses of the species (mass_C_, mass_R_, mass_E_, mass_H_, and mass_A_ in grams); and daily proportion of each predator's diet that is adult bird, hatchling, or egg (preypref_A,C_, preypref_H,C_, preypref_H,R_, and preypref_E,R_) as follows:

(4a) $$p_{A,C}=\frac{{\mathrm{metdemand}}_{C}\left[\frac{\mathrm{kJ}}{\mathrm{cat}\times \mathrm{day}}\right]{\mathrm{preypref}}_{A,C}}{{\mathrm{mass}}_{A}\left[\frac{g}{\mathrm{bird}}\right]\times {\mathrm{energy}}_{A}\left[\frac{\mathrm{kJ}}{g}\right]},\;$$

(4b) $$p_{H,C}=\frac{{\mathrm{metdemand}}_{C}\left[\frac{\mathrm{kJ}}{\mathrm{cat}\times \mathrm{day}}\right]{\mathrm{preypref}}_{H,C}}{{\mathrm{mass}}_{H}\left[\frac{g}{\mathrm{bird}}\right]\times {\mathrm{energy}}_{H}\left[\frac{\mathrm{kJ}}{g}\right]}\;,$$

(4c) $$p_{H,R}=\frac{{\mathrm{metdemand}}_{R}\left[\frac{\mathrm{kJ}}{\mathrm{rat}\times \mathrm{day}}\right]{\mathrm{preypref}}_{H,R}}{{\mathrm{mass}}_{H}\left[\frac{g}{\mathrm{bird}}\right]\times {\mathrm{energy}}_{H}\left[\frac{\mathrm{kJ}}{g}\right]}\;,$$

(4d) $$p_{E,R}=\frac{{\mathrm{metdemand}}_{R}\left[\frac{\mathrm{kJ}}{\mathrm{rat}\;\times \mathrm{day}}\right]{\mathrm{preypref}}_{E,R}}{{\mathrm{mass}}_{E}\left[\frac{g}{\mathrm{egg}}\right]\times {\mathrm{energy}}_{E}\left[\frac{\mathrm{kJ}}{g}\right]}\;,$$

where A is adult birds, H is hatchlings, and E is eggs. Parameter values are in Table 1. We used published estimates for the parameters from Christmas Island wherever possible. If these were not available, we used estimates of the same species from other locations and energetic limitations on metabolic rates (Table 1). Estimates for the prey preferences for cats were taken from a scat study from 2 Galapagos islands, where birds constituted 38.2% of their diet (Konecny, 1987). In a study on rat diet on Stewart Island, New Zealand, birds made up 8% of the stomach contents and occurred in 25% of samples. We used this information to average the bird content in rat diet over all rats and to calculate a preypref_H,R_ of 0.02. Because we could not find an estimate for preference of eating eggs, we assumed eggs are twice as likely to be eaten as live animals because they are immobile and not constantly guarded by parents. To calculate the metabolic demands of the predator species, we used equations derived from field studies of metabolic rates of the animal taxa carnivora and Rodentia (Nagy et al., 1999) (Table 1).

**TABLE 1:** Estimates of parameter values and their origin in a model of total predation on red‐tailed tropicbirds

Parameter	Description	Unit	Mean	Range	Source
*N*_A,0_	Population size of red‐tailed tropicbirds	individuals	2800	†	Stokes, 1988
*T*_I_	Duration of incubation time	days	42	–	
*T*_H_	Duration of juveniles as hatchlings	days	90	–	
*T*_B_	Duration of breeding season	days	122	–	
*T*_mat_	Time juveniles spend of the island	days	*α*_R_ – *T*_B_/365	–	
*α*_M_	Age to reproductive maturity	years	3	–	del Hoyo et al., 1992
*α*_R_	Life span	years	15	–	
*β*	Eggs laid per adult prey per breeding season	number	$0.5\times (\frac{\alpha_{M}-\alpha_{R}}{\alpha_{M}})$	†	
*ν*	Proportion of viable eggs	proportion	0.99	†	No literature estimate available
*μ*_A_	Annual natural mortality rate of adults	proportion	0.125	0.10–0.15	Schreiber & Schreiber, 1993; Schreiber et al, 2001, 2004
*μ*_J_	Annual natural mortality rate of juveniles	proportion	0.20	†	Schreiber et al. 2004
*μ*_H_	Natural mortality rate of hatchlings over breeding season	proportion	$1-{\left(1-\mu_{j}\right)}^{\frac{T_{H}}{365-T_{I}}}$	†	See *μ*_J_
preypref_A,C_	Proportion of adult bird in cat diet	proportion	0.382	†	Konecny, 1987
preypref_H,C_	Proportion of hatchling in cat diet	proportion	0.382	†	
preypref_H,R_	Proportion of hatchling in rat diet	proportion	0.02	†	Gales, 1982
preypref_E,R_	Proportion of egg in rat diet	proportion	0.04	†	No literature estimate available
mass_C_	Cat mass	g/individual	3250	2000–4500	Moseby et al., 2015
mass_R_	Rat mass	g/individual	132	15–250	CI data; Willacy, personal communication
mass_A_	Adult bird mass	g/individual	700	600–800	del Hoyo et al., 1992
mass_H_	Hatchling bird mass	g/individual	$\frac{{\mathrm{mass}}_{A}}{2}$	300–400	
mass_E_	Egg mass	g/individual	66.2	†	Lobel et al., 2012
energy_A_	Energy content of bird meat	kJ/g	10.9	†	Matias & Catry, 2008
energy_H_	Energy content of bird meat	kJ/g	10.9	†	
energy_E_	Energy content of egg	kJ/g	29	†	
metdemand_C_	Daily metabolic demand in feral cats	kJ∙individual^–1^∙day^–1^	$1.67\times {\mathrm{mass}}_{C}^{0.869}$	†	Nagy et al., 1999
metdemand_R_	Daily metabolic demand in wild rats	kJ∙individual^–1^∙day^–1^	$5.48\times {\mathrm{mass}}_{R}^{0.712}$	†	

*Note*: Symbol “†” represents no range around the mean estimate of the parameter value, so 20% variation assumed. Symbol “–” represents no range assumed for temporal parameters.

### Model application

A model of reproduction ratio was produced by substituting Equations (2)–(4) into Equation (1), and it was used to define a predator phase space of predator abundances for which the reproductive ratio of the red‐tailed tropicbird population was predicted by our model to remain stable at different certainties. By setting *η* to 1, substituting Equations (2) and (3) into Equation (1) yielded an equation that predicts, for a given *N*_cats_, the maximum number of rats *N*_rats_ for which the tropic bird population is sustainable (i.e., η≥1):

(5) $$\;N_{\mathrm{rats}}=\mathrm{critical}\;\mathrm{rats}-\mathrm{cat}\;\mathrm{equivalence}\;\times \;N_{\mathrm{cats}},$$

where critical rats (Equation 6) is the maximum number of rats such that *η* = 1 in absence of any cats, and cat equivalence (Equation 7) is the number of rats that have the equivalent effect on *η* as 1 cat. The model does not predict the dynamic interactions between cat and rat populations, but rather captures how the size of these 2 threats together affect the red‐tailed tropicbird population: the metrics critical rats and cat equivalence in Equation (5) can be derived directly from Equations (1)–(3):

(6) $$\mathrm{critical}\;\mathrm{rats}=\frac{N_{A,0}\nu \beta \left(1-\mu_{H}\right)-\eta N_{A,0}\mu_{A}\;{\left(1-\mu_{J}\right)}^{-T\mathrm{mat}}}{p_{H,R}T_{H}+\nu \left(1-\mu_{H}\right)p_{E,R}T_{I}}$$

and

(7) $$\mathrm{cat}\;\mathrm{equivalence}=\frac{p_{H,C}T_{H}+p_{A,C}\eta \;T_{B}{\left(1-\mu_{J}\right)}^{-T\mathrm{mat}}}{p_{H,R}T_{H}+\nu \left(1-\mu_{H}\right)\;p_{E,R}T_{I}}.$$

The values of critical rats and cat equivalence were calculated from Equations (6) and (7) with parameter values in Equations (4a)–(4d) and Table 1. A critical cats metric (i.e., the maximum size of cat population for which η=1 in absence of any rats) was derived by rearranging Equation (5) for *N*_rats_ = 0 and given by criticalcats=criticalrats/catequivalence.

### Parameter uncertainty

Many of the estimates for the parameter values were either not available for the study site, did not come with an estimate of variation, or both. To account for uncertainty in the estimates of parameter values for which variation was unknown, we assumed that each model parameter was normally distributed and had a standard deviation that corresponded to 20% around the reported mean estimate of the parameter value (Table 1). This choice is based on the average variation of the parameters for which variation estimates were available in the literature (see Table 1). Normal distributions for all model parameters were assumed to be independent of each other (i.e., zero covariance). We estimated probability distributions for the predictions of cat equivalence, critical rats, and other relevant model outputs by using the Monte Carlo method (Xiao et al., 2017). The output distributions were summarized via calculation of their medians and 68% and 95% central credible intervals. These quantities characterized the uncertainty in the predictions of our model.

**FIGURE 2:**
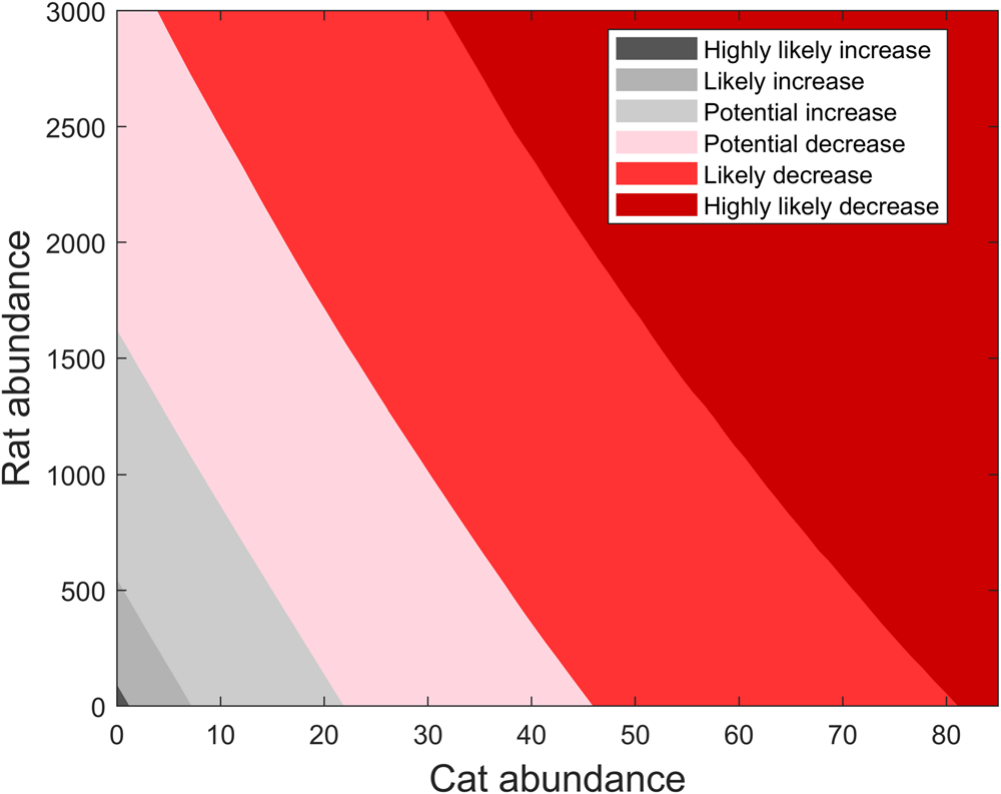
Associated behavior of the reproduction ratio of the population of red‐tailed tropicbirds on Christmas Island at different relative abundances of predators (feral cats and rats). The border between potential decline and potential increase represents the median estimate of predator abundances at which a reproduction ratio (*η*) of 1 is maintained. To represent uncertainty, upper and lower bounds for the 68% and 95% credible interval (CI) are shown for the predator abundances that yield *η* = 1 as borders between shaded areas. That is, at each cat abundance value, the rat abundances that correspond to the upper and lower bounds on the 68% CI for *η* = 1 are indicated by the potential or likely borders for tropicbird decrease and increase, respectively. Similarly, at each cat abundance value, the rat abundances that correspond to the upper and lower bounds on the 95% CI for *η* = 1 are indicated by the likely or highly likely borders for tropicbird decrease and increase, respectively.

We also displayed the uncertainty in model outputs in cat–rat phase space. For each parameter combination drawn, there was a line demarking the critical threshold for rats as a function of the number of cats in the system (see Equation 5). For cat and rat populations above the line, the bird population was threatened; below the line, the bird population was not threatened. The *y*‐intercept of the line was critical rats, and the slope was negative cat equivalence (see Equation 5). In the above uncertainty analysis, we generated a line for each parameter combination. With all lines in hand, for every cat population size, we evaluated the corresponding many estimates of maximum rat population sizes obtained from each of the lines. Instead of plotting all these lines, we identified rat abundances that specify the median and bound the middle 68% and 95% of the maximum rat population sizes obtained from all of these (unplotted) lines. Repeating this process for every cat population size generated the boundaries of the regions in Figure 2.

### Sensitivity of cat equivalence

To analyze the sensitivity of the cat equivalence metric to parameter uncertainty, we applied a strong variation (i.e., 75%) to each parameter separately and calculated the change in estimate of cat equivalence for each parameter. Although most of the literature estimates of parameter values varied far less than 75%, choosing a large variation helped in detection of the relative sensitivity of the estimate of cat equivalence to individual parameters. This sensitivity analysis was used to identify which parameters had the largest effect on the cat equivalence metric. The parameters *N*_A,0_, *β*, and *μ*_A_ were excluded from this sensitivity analysis because cat equivalence did not depend on these parameters (Equation 7).

## RESULTS

### Critical predator abundances and cat equivalence

Our model predicted that the red‐tailed tropicbird population has equal probability of increasing or decreasing (actual population trajectory dependent on the true, but unknown, values of the model parameters) in the presence of approximately 21 cats in absence of rats or approximately 1607 rats in absence of cats. These 2 values appear at the intersection of Figure 2’s curve that separates pink and light gray regions and the horizontal and vertical axes. The values could also be interpreted as thresholds, that is, predator abundances above these values would increase the probability of a declining tropicbird population and lower predator abundances would increase the probability of an expanding tropicbird population.

More generally, if cats and rats were to co‐occur, there would be a 50–84%, 84–97.5%, and >97.5% probability of tropicbird population decline if the number of cats and rats were to fall in a pink, light red, or dark red region, respectively, of Figure 2. Conversely, if the present number of cats and rats was to fall in a light gray, medium gray, or dark gray region of Figure 2, there would be a 50–84%, 84–97.5%, and >97.5% probability of tropicbird population expansion, respectively. All these probabilities were calculated based on the assumption that the probability distributions used for model parameters were correct. These probabilities provided a range of possible outcomes for the tropicbird population based on the current number of cats and rats.

### Parameter sensitivity

**FIGURE 3:**
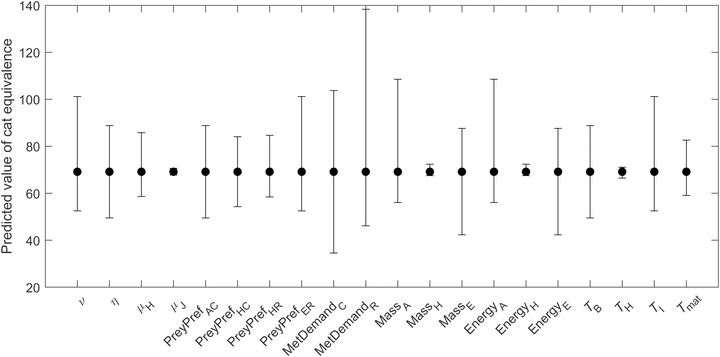
Sensitivity of cat equivalence to 75% uncertainty in the estimates of each parameter value in a model of total predation on red‐tailed tropicbirds. Cat equivalence is independent of parameters *A*_0_, *β*, and *μ*_A_; hence, they do not appear in the plot. Parameters are defined in Table 1.

Metabolic demand of rats had the strongest individual effect on the cat equivalence (Figure 3). The second most influential parameters were mass and energy of adult birds, followed by the metabolic demands of cats. Hatchling parameters (e.g., *T*_H_, mass_H_, energy_H_) had very little effect on the predictions of cat equivalence (Figure 3).

### Distribution of estimates for cat equivalence and critical rats

The uncertainty in the estimates of parameter values resulted in strongly right‐skewed distributions of cat equivalence and critical rats (Appendix S1a). Cat equivalence ranged from around 10 to 410 (median around 77). Critical rat numbers varied widely, from 0 to 13,000 (median 1407) (Appendix S1b).

## DISCUSSION

Predicting the consequences of management interventions in ecosystems that face multiple threats is difficult because they exhibit complex dynamics and knowledge to parameterize these models is often scarce (Geary et al., 2020). Instead of predicting the population state of a threatened species after a management action (as in, e.g., Baker et al. [2017], Bode et al. [2015], Dambacher et al. [2003], and Han et al. [2020]), our approach identified the levels of threat that could lead to undesired population trajectories in a threatened species and could thereby trigger a management decision. For example, on Christmas Island, our methods would allow managers to determine whether the current abundance of predators threatens the red‐tailed tropicbird population; whether managing cats alone is sufficient to protect the birds; and whether and how much additional rat control is necessary. The last point is particularly useful to managers given the costs of multispecies eradications can be significantly higher single‐species eradications (Baker et al., 2020), and, because rat eradications can be difficult (Holmes et al., 2015), managers may choose to eradicate only cats. Our estimate of critical rats served as the quantitative level of rat abundances that should not be exceeded to maintain desired bird reproduction.

Theoretical studies show that the rat populations may increase under certain conditions when managing cats on Christmas Island (Baker et al., 2020; Han et al., 2020). It is useful to know how many additional rats would lead to an undesired bird reproduction rate. This abundance would then trigger required rat control. By presenting 1 predator in the units of another, the estimate of cat equivalence provided a quantitative target of how much the rat abundances could increase (with every cat removed) before leading to an undesired bird reproduction rate. For example, a mesopredator release of <77 rats per eradicated cat was <50% likely to threaten the population trajectory of red‐tailed tropicbirds (Figure 2). The lower the rat value, the lower the probability of an undesired reproduction rate. Thus, when controlling cats on Christmas Island, a simultaneous assessment of rat abundances could determine whether rats need to be managed as well and allow swift action when rat numbers increase. Using rat abundances as a decision trigger allows managers to act even before rats negatively affect the population of red‐tailed tropicbirds. Sometimes, complete eradications of cats are difficult to achieve (Campbell et al., 2011); hence, if cats remain, the critical number of rats that allow the desired reproduction ratio of the red‐tailed tropicbird would be lower. In case of remaining cats, the cat equivalence thus serves as a quantitative estimate for the reduction of the critical rats.

Our approach delivers decision triggers with an uncertainty estimate. Data‐scarce systems are characterized by large parametric uncertainty that is amplified in model outputs, and our model was not immune to this. Although the large uncertainty in our model outputs may deter application of the method, conservation decision‐making and management occurs even under high, albeit often unstated, uncertainty (McCarthy et al., 2014). Modeling and risk analysis should prepare conservation managers for the presence and the impact of uncertainty (Canessa et al., 2016; Lechner et al., 2014; McCarthy et al., 2014). Providing quantitative estimates of the uncertainty of a management intervention allows the decision maker to make informed and transparent decisions (Canessa et al., 2016). By presenting uncertainty bounds of the decision triggers in our case study, we provided managers with a tool to assess their risk tolerance when deciding about the predator abundances that trigger management interventions. In the case of risk‐averse decision makers, they can decide to not proceed with the management intervention and instead gather more information to improve the estimates and reduce uncertainty. A prudent next step would be to validate the estimates with field data to determine whether the predictions of cat and rat abundances, which maintain a neutral reproductive ratio of tropicbirds, are within the uncertainty bounds.

We developed the model with biologically meaningful parameters (Adams et al., 2017) (i.e., parameters that are stable, physically interpretable, and transferable to other contexts). Biologically meaningful parameters can be measured in the field (such as prey preferences, metabolic demand, mass, etc.) and updated when more information becomes available. By assessing the sensitivity of cat equivalence, we provided a focal point for research to reduce the uncertainties in the estimates. The metabolic demands of rats and cats (which included the mass of rats and cats) and the mass and energy of adult birds influenced the estimates of cat equivalence most strongly. Studying these values on Christmas Island could help reduce the uncertainty in the cat equivalence and probably also in the estimates of critical predator abundances. To do so, cats and rats could be caught alive to assess their weights, and metabolic demands could be measured using direct or indirect calorimetry (Kaiyala & Ramsey, 2011). We could have instead used parameters that model the interaction more directly, for example, the interaction strength between predators and red‐tailed tropicbirds. However, it is difficult to measure individual interaction rates because they imply a mass action assumption, where the rate of change of a species is proportional to the product of abundance of the species with the abundance of the interaction partner species (Baker et al., 2020). We divided interactions into independently measurable things, such as energy contents and energy requirements, to allow the possibility of measuring and updating the estimates of parameter values. Although models with biologically meaningful parameters may not have the best fit, they can be updated, used beyond the chosen system, and create connections between research and management (Adams et al., 2017).

Although our approach is most easily applied to small ecosystems with 1 threatened component and 2 threats, in reality many more ecosystem components can influence such a small system. For example, the network of species interacting on Christmas Island include a number of other species (Han et al., 2020). Further, red‐tailed tropicbirds can be affected by other threatening processes such as extreme weather events (Hennicke & Flachsbart, 2009) and lack of food resources (Schreiber, 1994). Adding more components to the model, however, may not necessarily improve model performance (Arhonditis & Brett, 2004) and may increase the uncertainty in outcomes. We focused our modeling efforts on the species at risk and the 2 threatening processes to reflect current management decisions (i.e., eradication of cats and rats) and to avoid adding more complexity to the model.

One possible extension for our approach, however, could be to gather information on costs and success rates of management interventions to develop a cost‐efficacy framework for management decisions and use it to inform planning for management effort. For example, if reducing cat populations by 50% costs twice as much as reducing rats by 50%, but each cat is worth 77 rats, then our cat equivalence metric suggests that removing cats would be the most cost‐effective strategy, but if cat equivalence is 30 then removing rats would be more effective.

Our approach could be applied to other cases in which multiple biotic or abiotic or both biotic and abiotic threats affect the population of a threatened species. For example, hispid cotton rats (*Sigmodon hispidus*), a rodent native to parts of the Americas, can be directly killed by fire, yet fire can also indirectly increase predation pressure by burning cover vegetation (Conner et al., 2011). Prescribed burning of fire‐maintained longleaf pine (*Pinus palustris*) forests can dramatically decrease cotton rat populations, but predator control may mitigate these effects. To apply our approach to this system, an appropriate population indicator would need to account for direct and indirect mortalities due to fire, as well as natural birth and mortality rates and baseline predation rates without fire. Our threat equivalence approach could express the impact of prescribed fire as the threat from predators and thereby inform decisions about potential management actions. Knowing the decision triggers allows managers to assess whether predator abundances could affect the rat population in the case of a prescribed fire. Managers could then decide to either delay burning or decrease predator numbers if immediate burning is required.

Invasive species are among the 5 key drivers of ecosystem change (IPBES, 2019) and represent one of the largest challenges to global biosphere integrity (Steffen et al., 2015). The unintended consequences of managing only 1 of several invasive species is widely documented (e.g., Prior et al., 2018; Rayner et al., 2007; Wittmer et al., 2013). Although traditional approaches can estimate potential success of a specific management intervention, which helps in the selection among management interventions, it may not necessarily provide guidance on how to react in the case of an undesired outcome. We provide managers with a decision tool to assess critical abundances of threats and react to changes in them. Estimating threat equivalence and shifting from predicting the possible future under different scenarios to assessing the conditions under which outcomes of concern are likely may have applications beyond conservation.

## Supporting information

Fig. S1 Uncertainty in a) cat equivalence and b) critical rats calculated using the Monte Carlo method. Estimates of the parameter values were sampled from truncated normal distributions within the parameter range. If no estimates of range for a parameter were available, we assumed a 20% variation.

## References

[cobi13916-bib-0001] Adams, M. P. , Collier, C. J. , Uthicke, S. , Ow, Y. , Langlois, L. , & O'Brian, K. R. (2017). Model fit versus biological relevance: Evaluating photosynthesis‐temperature models for three tropical seagrass species. Scientific Reports, 7(1), 1–12. 28051123 10.1038/srep39930 PMC5209739

[cobi13916-bib-0002] Adams, M. P. , Sisson, S. A. , Helmstedt, K. J. , Baker, C. M. , Holden, M. H. , Plein, M. , Holloway, J. , Mengersen, K. L. , & McDonald‐Madden, E. (2020). Informing management decisions for ecological networks, using dynamic models calibrated to noisy time‐series data. Ecology Letters, 23(4), 607–619. 31989772 10.1111/ele.13465

[cobi13916-bib-0003] Algar, D. D. , & Hamilton, N. , (2014). Report on stage 2(d) of the Christmas Island cat and black rat management plan . Western Australian Department of Parks and Wildlife.

[cobi13916-bib-0004] Arhonditsis, G. B. , & Brett, M. T. (2004). Evaluation of the current state of mechanistic aquatic biogeochemical modeling. Marine Ecology Progress Series, 271, 13–26.

[cobi13916-bib-0005] Baker, C. M. , Gordon, A. , & Bode, M. (2017). Ensemble ecosystem modelling for predicting ecosystem response to predator reintroduction. Conservation Biology, 31(2), 376–384. 27478092 10.1111/cobi.12798

[cobi13916-bib-0006] Baker, C. M. , Holden, M. H. , Plein, M. , McCarthy, M. A. , & Possingham, H. P. (2018). Informing network management using fuzzy cognitive maps. Biological Conservation, 224, 122–128.

[cobi13916-bib-0007] Baker, C. M. , Plein, M. , Shaikh, R. , & Bode, M. (2020). Simultaneous invasive alien predator eradication delivers the best outcomes for protected island species. Biological Invasions, 22(3), 1085–1095.

[cobi13916-bib-0008] Beeton, B. , Burbridge, A. , Grigg, G. , Harrison, P. , How, R. , Humphreys, B. , McKenzie, N. , & Woinarski, J. (2010). Final report of the Christmas Island expert working group to the Minister for Environment Protection, Heritage, and the Arts . Department of the Environment, Protection, Heritage, and the Arts.

[cobi13916-bib-0010] Bode, M. , Baker, C. M. , & Plein, M. (2015). Eradicating down the food chain: Optimal multispecies eradication schedules for a commonly encountered invaded island ecosystem. Journal of Applied Ecology, 52(3), 571–579.

[cobi13916-bib-0012] Campbell, K. J. , Harper, G. , Algar, D. , Hanson, C. C. , Keitt, B. S. , & Robinson, S. (2011). Review of feral cat eradications on islands. In C. R. Veitch , M. N. Clout , & D. R. Towns (Eds.), Island invasives: Eradication and management (pp. 37–46). IUCN.

[cobi13916-bib-0013] Canessa, S. , Ewen, J. G. , West, M. , McCarthy, M. A. , & Walshe, T. V. (2016). Stochastic dominance to account for uncertainty and risk in conservation decisions. Conservation Letters, 9(4), 260–266.

[cobi13916-bib-0014] Conner, L. M. , Castleberry, S. B. , & Derrick, A. M. (2011). Effects of mesopredators and prescribed fire on hispid cotton rat survival and cause‐specific mortality. The Journal of Wildlife Management, 75(4), 938–944.

[cobi13916-bib-0017] Dambacher, J. M. , Li, H. W. , & Rossignol, P. A. (2003). Qualitative predictions in model ecosystems. Ecological Modelling, 161(1–2), 79–93.

[cobi13916-bib-0018] del Hoyo, J. , Elliott, A. , & Sargatal, J. (Eds.). (1992). Handbook of the birds of the world ‐ Volume 1: Ostrich to ducks. Lynx Edicions.

[cobi13916-bib-0020] Doherty, T. S. , Glen, A. S. , Nimmo, D. G. , Ritchie, E. G. , & Dickman, C. R. (2016). Invasive predators and global biodiversity loss. Proceedings of the National Academy of Sciences of the United States of America, 113(40), 11261–11265. 27638204 10.1073/pnas.1602480113 PMC5056110

[cobi13916-bib-0021] Fleet, R. R. (1974). The red‐tailed tropicbird on Kure Atoll. Ornithological Monographs, 16, 1–64.

[cobi13916-bib-0023] Gales, R. (1982). Age‐ and sex‐related differences in diet selection by *Rattus rattus* on Stewart Island, New Zealand. New Zealand Journal of Zoology, 9, 463–466.

[cobi13916-bib-0024] Geary, W. L. , Bode, M. , Doherty, T. S. , Fulton, E. A. , Nimmo, D. G. , Tulloch, A. I. , Tulloch, V. J. , & Ritchie, E. G. (2020). A guide to ecosystem models and their environmental applications. Nature Ecology & Evolution, 4(11), 1459–1471. 32929239 10.1038/s41559-020-01298-8

[cobi13916-bib-0025] Han, Y. , Kristensen, N. P. , Buckley, Y. M. , Maple, D. J. , West, J. , & McDonald‐Madden, E. (2020). Predicting the ecosystem‐wide impacts of eradication with limited information using a qualitative modelling approach. Ecological Modelling, 430, 109122.

[cobi13916-bib-0026] Hennicke, J. C. , & Flachsbarth, K. (2009). Effects of cyclone Rosie on breeding red‐tailed tropicbirds *Phaeton rubricauda* on Christmas Island. Marine Ornithology, 37, 175–178.

[cobi13916-bib-0027] Holmes, N. , Griffiths, R. , Pott, M. , Alifano, A. , Will, D. , Wegmann, A. , & Russell, J. (2015). Factors associated with rodent eradication failure. Biological Conservation, 185, 8–16.

[cobi13916-bib-0028] Hughes, B. , Dickey, R. , & Reynolds, S. (2019). Predation pressures on sooty terns by cats, rats and common mynas on Ascension Island in the South Atlantic. In C. R. Veitch , M. N. Clout , A. R. Martin , J. C. Russell , & C. J. West (Eds.), Island invasives: Scaling up to meet the challenge: Proceedings of the International Conference on Island Invasives (Occasional Paper SSC no. 62, pp. 295–301). IUCN.

[cobi13916-bib-0029] Intergovernmental Science‐Policy Platform on Biodiversity and Ecosystem Services (IPBES) . (2019). Global assessment report on biodiversity and ecosystem services of the Intergovernmental Science‐Policy Platform on Biodiversity and Ecosystem Services . IPBES Secretariat.

[cobi13916-bib-0030] Ishii, N. (2006). *A survey of Red‐tailed Tropicbird* Phaethon rubricauda *at the Sitting Room and Rumah Tinggi, Christmas Island, April–July 2006* . Parks Australia North, Christmas Island.

[cobi13916-bib-0031] Jachowski, D. S. , Butler, A. , Eng, R. Y. , Gigliotti, L. , Harris, S. , & Williams, A. (2020). Identifying mesopredator release in multi‐predator systems: A review of evidence from North America. Mammal Review, 50(4), 367–381.

[cobi13916-bib-0032] James, D. J. , & McAllan, I. A. (2014). The birds of Christmas Island, Indian Ocean: A review. Australian Field Ornithology, 31, S1–S175.

[cobi13916-bib-0033] Kaiyala, K. J. , & Ramsay, D. S. (2011). Direct animal calorimetry, the underused gold standard for quantifying the fire of life. Comparative Biochemistry and Physiology Part A: Molecular & Integrative Physiology, 158(3), 252–264. 10.1016/j.cbpa.2010.04.013 PMC3920988 20427023

[cobi13916-bib-0034] Konecny, M. J. (1987). Food habits and energetics of feral house cats in the Galápagos Islands. Oikos, 50, 24–32.

[cobi13916-bib-0035] Lechner, A. M. , Raymond, C. M. , Adams, V. M. , Polyakov, M. , Gordon, A. , Rhodes, J. R. , Mills, M. , Stein, A. , Ives, C. D. , & Lefroy, E. C. (2014). Characterizing spatial uncertainty when integrating social data in conservation planning. Conservation Biology, 28(6), 1497–1511. 25382071 10.1111/cobi.12409

[cobi13916-bib-0037] Lobel, P. , Schreiber, E. A. , McGloskey, G. , & O'Shea, L. (2012). An ecological assessment of Johnston Atoll. Washington Group International.

[cobi13916-bib-0038] Matias, R. , & Catry, P. (2008). The diet of feral cats at New Island, Falkland Islands, and impact on breeding seabirds. Polar Biology, 31(5), 609–616.

[cobi13916-bib-0039] Matlab . (2021). Version 9.10 (R2021a). The MathWorks Inc.

[cobi13916-bib-0039a] McCarthy, M. A. (2014). Contending with uncertainty in conservation management decisions. Annals of the New York Academy of Sciences, 1322(1), 77–91. 25138920 10.1111/nyas.12507 PMC4312896

[cobi13916-bib-0040] Moseby, K. , Peacock, D. , & Read, J. (2015). Catastrophic cat predation: A call for predator profiling in wildlife protection programs. Biological Conservation, 191, 331–340.

[cobi13916-bib-0041] Nagy, K. A. , Girard, I. A. , & Brown, T. K. (1999). Energetics of free ranging mammals, reptiles, and birds. Annual Review of Nutrition, 19(1), 247–277. 10.1146/annurev.nutr.19.1.247 10448524

[cobi13916-bib-0042] Pearson, D. E. , Clark, T. J. , & Hahn, P. G. (2022). Evaluating unintended consequences of intentional species introductions and eradications for improved conservation management. Conservation Biology, 36(1), e13734. 33734489 10.1111/cobi.13734 PMC9291768

[cobi13916-bib-0043] Perryman, H. A. , Hansen, C. , Howell, D. , & Olsen, E. (2021). A review of applications evaluating fisheries management scenarios through marine ecosystem models. Reviews in Fisheries Science & Aquaculture, 29, 800–835.

[cobi13916-bib-0044] Peterson, K. A. , Barnes, M. D. , Jeynes‐Smith, C. , Cowen, S. , Gibson, L. , Sims, C. , Baker, C. M. , & Bode, M. (2021). Reconstructing lost ecosystems: A risk analysis framework for planning multispecies reintroductions under severe uncertainty. Journal of Applied Ecology, 58(10), 2171–2184.

[cobi13916-bib-0045] Prior, K. M. , Adams, D. C. , Klepzig, K. D. , & Hulcr, J. (2018). When does invasive species removal lead to ecological recovery? Implications for management success. Biological Invasions, 20(2), 267–283.

[cobi13916-bib-0046] Prugh, L. R. , Stoner, C. J. , Epps, C. W. , Bean, W. T. , Ripple, W. J. , Laliberte, A. S. , & Brashares, J. S. (2009). The rise of the mesopredator. Bioscience, 59(9), 779–791.

[cobi13916-bib-0047] Rayner, M. J. , Hauber, M. E. , Imber, M. J. , Stamp, R. K. , & Clout, M. N. (2007). Spatial heterogeneity of mesopredator release within an oceanic island system. Proceedings of the National Academy of Sciences of the United States of America, 104(52), 20862–20865. 18083843 10.1073/pnas.0707414105 PMC2409232

[cobi13916-bib-0048] Rendall, A. R. , Sutherland, D. R. , Baker, C. M. , Raymond, B. , Cooke, R. , & White, J. G. (2021). Managing ecosystems in a sea of uncertainty: Invasive species management and assisted colonizations. Ecological Applications, 31(4), e02306. 33595860 10.1002/eap.2306

[cobi13916-bib-0049] Reum, J. C. , Kelble, C. R. , Harvey, C. J. , Wildermuth, R. P. , Trifonova, N. , Lucey, S. M. , McDonald, P. S. , & Townsend, H. (2021). Network approaches for formalizing conceptual models in ecosystem‐based management. ICES Journal of Marine Science, 78(10), 3674–3686.

[cobi13916-bib-0050] Ritchie, E. G. , & Johnson, C. N. (2009). Predator interactions, mesopredator release and biodiversity conservation. Ecology Letters, 12(9), 982–998. 19614756 10.1111/j.1461-0248.2009.01347.x

[cobi13916-bib-0051] Schreiber, E. A. , & Schreiber, R. W. (1993). *Red‐tailed tropicbird* (Phaethon rubricauda). The Birds of North America Online.

[cobi13916-bib-0052] Schreiber, E. A. (1994). El Niño‐Southern Oscillation effects on provisioning and growth in red‐tailed tropicbirds. Colonial Waterbirds, 17, 105–119.

[cobi13916-bib-0053] Schreiber, E. A. , Doherty, P. F., Jr. , & Schenk, G. A. (2001). Effects of a chemical weapons incineration plant on red‐tailed tropicbirds. The Journal of Wildlife Management, 65(4), 685–695.

[cobi13916-bib-0054] Schreiber, E. A. , Doherty, P. F. , & Schenk, G. A. (2004). Dispersal and survival rates of adult and juvenile red‐tailed tropicbirds (*Phaethon rubricauda*) exposed to potential contaminants. Animal Biodiversity and Conservation, 27, 1–11.

[cobi13916-bib-0055] Shannon, G. , Matthews, W. S. , Page, B. R. , Parker, G. E. , & Smith, R. J. (2009). The affects of artificial water availability on large herbivore ranging patterns in savanna habitats: A new approach based on modelling elephant path distributions. Diversity and Distributions, 15(5), 776–783.

[cobi13916-bib-0056] Smith, R. K. , Pullin, A. S. , Stewart, G. B. , & Sutherland, W. J. (2010). Effectiveness of predator removal for enhancing bird populations. Conservation Biology, 24(3), 820–829. 20067492 10.1111/j.1523-1739.2009.01421.x

[cobi13916-bib-0057] Sommerfeld, J. , Stokes, T. , & Baker, G. B. (2015). Breeding success, mate‐fidelity and nest‐site fidelity in Red‐tailed Tropicbirds (*Phaethon rubricauda*) on Christmas Island, Indian Ocean. Emu‐Austral Ornithology, 115(3), 214–222.

[cobi13916-bib-0058] Steffen, W. , Richardson, K. , Rockström, J. , Cornell, S. E. , Fetzer, I. , Bennett, E. M. , Biggs, R. , Carpenter, S. , De Vries, W. , De Wit, C. , Folke, C. , Gerten, D. , Heinke, J. , Mace, G. M. , Persson, L. M. , Ramanathan, V. , Reyers, B. , & Sörlin, S. (2015). Planetary boundaries: Guiding human development on a changing planet. Science, 347(6223), 1259855. 25592418 10.1126/science.1259855

[cobi13916-bib-0060] Stokes, T. (1988). A review of the birds of Christmas Island, Indian Ocean (Occasional Paper 16). Australian National Parks and Wildlife Service.

[cobi13916-bib-0061] Wittmer, H. U. , Elbroch, L. M. , & Marshall, A. J. (2013). Good intentions gone wrong: Did conservation management threaten Endangered huemul deer *Hippocamelus bisulcus* in the future Patagonia National Park? Oryx, 47(3), 393–402.

[cobi13916-bib-0062] Xiao, M. , Adams, M. P. , Willis, A. , Burford, M. A. , & O'Brien, K. R. (2017). Variation within and between cyanobacterial species and strains affects competition: Implications for phytoplankton modelling. Harmful Algae, 69, 38–47. 29122241 10.1016/j.hal.2017.10.001

