# Supplementary material for: Modeling total predation to avoid perverse outcomes from cat control in a data‐poor island ecosystem
Source: Conserv Biol. 2022 Aug 24;36(5):e13916. doi: 10.1111/cobi.13916 (PMC9804458; doi:10.1111/cobi.13916)

Appendix

S1 Uncertainty in a) cat equivalence and b) critical rats calculated using the Monte Carlo method. Estimates of the parameter values were sampled from truncated normal distributions within the parameter range. If no estimates of range for a parameter were available, we assumed a 20% variation.

| a) | b) |
| --- | --- |


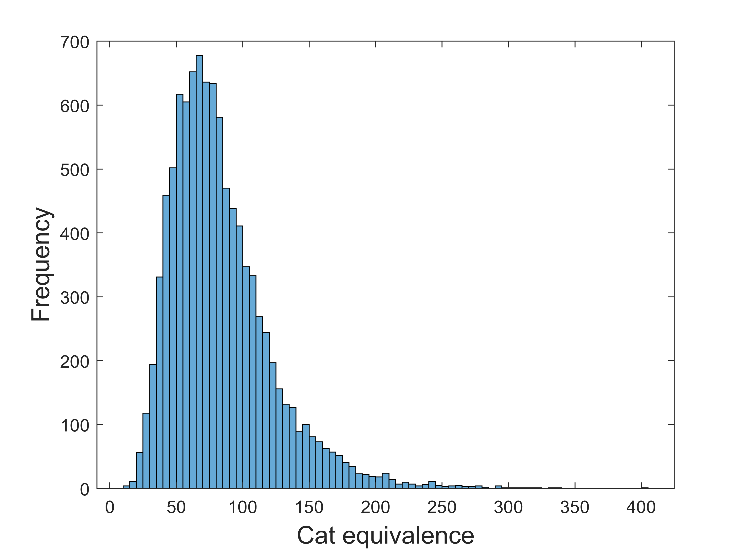

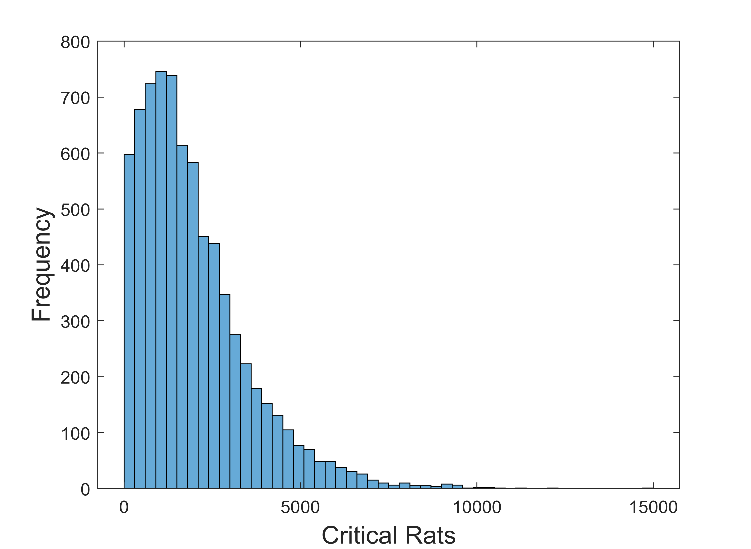

Supplement: Supplementary file 1 — Fig. S1 Uncertainty in a) cat equivalence and b) critical rats calculated using the Monte Carlo method. Estimates of the parameter values were sampled from truncated normal distributions within the parameter range. If no estimates of range for a parameter were available, we assumed a 20% variation. [file COBI-36-0-s001.docx]
